# The use of a novel ultrasound guidance system for real-time central venous cannulation: initial report on safety and efficacy

**DOI:** 10.1186/2036-7902-6-S1-A2

**Published:** 2014-01-31

**Authors:** RM Ferre, M Mercier

**Affiliations:** 1 Department of Emergency Medicine, Vanderbilt University, Nashville, TN, USA; 2 Department of Emergency Medicine, Palmetto Health Baptist, Columbia, SC, USA

## Background

Real-time ultrasound guidance is considered to be the standard of care for central venous access for non-emergent central lines. However, adoption has been slow in part because of the technical challenges and time required to acquire the skills to become proficient. The AxoTrack system (Soma Access Systems, Greenville, SC) is a novel ultrasound guidance system recently cleared for human use by the United States Food and Drug Administration (FDA).

## Objective

Quality assurance data was collected each time the AxoTrack system was used for central venous cannulation (CVC) to determine if the system was successful and to describe any complications from the procedure.

## Patients and methods

After FDA clearance, the AxoTrack system was released to three hospitals in the United States. Physicians and Nurse Practitioners who work in the Intensive Care Unit or Emergency Department and who place central venous catheters were trained to use the AxoTrack system. De-identified data about central lines placed in living patients with the AxoTrack system was prospectively gathered at each of the three hospitals for quality assurance purposes. After IRB approval, we consolidated the data for the first five months of use for retrospective review.

## Results

The AxoTrack system was used by 21 different health care providers in 50 consecutive patients undergoing CVC from September, 2012 to February, 2013. All patients had successful central venous cannulation with the guidance of the AxoTrack system. All but one patient had successful cannulation on the first site attempted. There were no reported complications, including pneumothorax, hemothorax, arterial puncture or arterial cannulation.

## Conclusion

The AxoTrack system was a safe and effective means of CVC that can be used by a variety of health care practitioners.

